# What is extinction research?

**DOI:** 10.1017/ext.2024.8

**Published:** 2024-04-15

**Authors:** John Alroy, Barry Brook

**Affiliations:** 1 School of Natural Sciences, Macquarie University, Sydney, NSW, Australia; 2 School of Natural Sciences, University of Tasmania, Hobart, TAS, Australia

The extinction of biological species is a modern concept that has been broadly understood for the last two centuries, following in the wake of Georges Cuvier’s pioneering research on fossils in the early 19th century (Rudwick, 1997). Today, there is little disagreement that most species of life to have ever lived on Earth are now extinct (Raup, 1990) and that extinction is currently occurring at a greatly elevated rapid pace (Lamkin and Miller, 2016). Conservation of Earth’s biodiversity is therefore of acute interest to all of humanity. Given this context, it is no coincidence that efforts to kickstart a new, interdisciplinary science of extinction were first made several decades ago (Lawton and May, 1995; Brook and Alroy, 2017).

One might therefore think that a straightforward characterisation of extinction research would be at hand. However, the field encompasses a range of studies from the purely biological to the cultural, necessitating a flexible definition. Furthermore, almost anything having to do with extinct or threatened species, ecosystems or cultural change could imaginably fall within this broad area. Indeed, one perspective is that all of palaeontology qualifies as extinction research because nearly all fossil organisms are extinct (Raup, 1990); and that all of conservation biology also qualifies because organisms are presumably worth conserving exactly when threatened with extinction (Soulé, 1985).

At the other extreme, one might argue that to qualify as such, extinction research must be concerned explicitly with documenting and explaining extinctions that have actually occurred. For example, a paper describing changes in foraminiferal community structure across the Cretaceous-Palaeogene (K-Pg) boundary might not qualify, because communities can change considerably even in the absence of extinction. Contrast this with Alvarez et al.’s (1980) argument for a bolide impact cause of the mass die-off event, which makes the extinction theme central and explicit. This illustrates the kind of research that sits obviously within our scope.

Yet both broad and fine brushes obscure the nuanced contributions from diverse fields that directly address extinction’s causes and consequences. In this brief essay, we therefore seek to strike a middle ground between broad and narrow conceptions. Our goal is to provide a definition that works for all disciplines. We outline not only easy cases on either end of the continuum of relevance, but also the often trans-disciplinary grey zone.

On a simple level, any research that cites extinction as a primary topic is by definition extinction research and therefore within the scope of *Cambridge Prisms: Extinction.* To cite an obvious example, the keystone Alvarez et al. (1980) paper mentioned earlier has the word ‘extinction’ in its title, and it was indeed every bit as relevant as the geochemistry underpinning its methods could be.

However, what goes in a paper title is not always a reliable indicator. Li et al. (2010) described an ‘extinct dinosaur’ with descriptive style, but fossil descriptions and morphological studies by themselves are not in scope, no matter how interesting they might be. Likewise, Delvaux et al. (2023) address a key topic of biological invasion (in trees), but this is also not ‘extinction research’, even though invasive species can of course cause extinction. The issue is that this kind of a paper may say little or nothing to draw the link.

Several common categories of research also fall in the grey zone. This does include many studies on biodiversity, such as those documenting gradients through time and space (Mannion et al., 2014 addressed both). Gradients have to come into existence somehow, and that somehow must involve processes such as speciation, extinction, immigration and extirpation (the localised loss of species). Yet those factors need to be disentangled to fall under the extinction research umbrella. At the opposite spatial scale, ecologists routinely publish compare-and-contrast analyses of diversity and composition in nearby ecological communities drawn from relatively natural and anthropogenically disturbed habitats. This sort of work is not really about extinction, which is quite unfortunate because little is often known about the fate of certain groups other than their ensemble diversity. Insects and most marine organisms, for example, often fall in that category, with relatively few species belonging to such taxa having been evaluated by the IUCN (Hochkirch et al., 2021).

Regardless, to be relevant to *Cambridge Prisms: Extinction*, something needs to be said in detail about the fact that an extinction or multiple extinctions did occur, is occurring, or is likely to occur – it is not sufficient just to note in a general way that a species is extinct or that it is threatened.

At the same time, it is important to emphasise that research on mass extinctions or individual species is only part of the domain of extinction research. Entities that are not even biological are also relevant, including cultural ones, as exemplified by our recent Special Issue. Languages are of key interest here. Amano et al. (2016) – a study of language extinction at the global scale – is a clear example. And so are biological entities that are not species per se: entire ecosystems may go extinct or be locally or regionally extirpated, and those cases are both intrinsically important and scientifically informative (Rodríguez et al., 2011).

We also must emphasise that extinction research goes not only beyond biology but beyond science as normally circumscribed. For instance, works in areas like philosophy and economics can fall within the domain (e.g. Swanson, 1999; Kaperbauer, 2017). Indeed, research outside of science that concerns the prospect of human extinction (Matheny, 2007) certainly has a home in this field as we conceive it – as does research that puts biological extinction in the context of humanity’s future (Dirzo et al., 2022).

In summary, we are eager to see extinction research expand as an interdisciplinary nexus, and for that to happen academics must be clear on what the nexus really is. We suggest that potential authors of a submission to *Cambridge Prisms: Extinction* consider the following criteria for evaluating whether an individual piece of research falls within the scope: (1) the system under consideration – biological or otherwise – must include a reproducing community of individuals that may collectively cease to exist; (2) the system is either extinct or at risk of extinction; (3) the research is concerned primarily with documenting or explaining this fact, not just with documenting general features of the system. Although perhaps abstract, we feel that this brief definition captures the spirit of the term ‘extinction research’, and we look forward to seeing its application as a spur to future investigation.
