# Supplementary material for: What is extinction research?
Source: Camb Prism Extinct. 2024 Apr 15;2:e13. doi: 10.1017/ext.2024.8 (PMC11895717; doi:10.1017/ext.2024.8)
Supplement: Peer review history

# Editor report: Recommendation: What is extinction research? — R0/PR2

**DOI:** 10.1017/ext.2024.8.pr2

**Affiliations:** Department of Geography and Geosciences, Friedrich-Alexander-Universitat Erlangen-Nurnberg, Germany

No accompanying comment.

# Editor report: Decision: What is extinction research? — R0/PR3

**DOI:** 10.1017/ext.2024.8.pr3

**Affiliations:** Department of Geography and Geosciences, Friedrich-Alexander-Universitat Erlangen-Nurnberg, Germany

No accompanying comment.
